# Coexistence of *tmexCD3-toprJ1b* tigecycline resistance genes with two novel *bla*_VIM-2_-carrying and *bla*_OXA-10_-carrying transposons in a *Pseudomononas asiatica* plasmid

**DOI:** 10.3389/fcimb.2023.1130333

**Published:** 2023-03-01

**Authors:** Qin Li, Qiao Chen, Shuang Liang, Wei Wang, Bingying Zhang, Alberto J. Martín-Rodríguez, Qinghua Liang, Feiyang Zhang, Ling Guo, Xia Xiong, Renjing Hu, Li Xiang, Yingshun Zhou

**Affiliations:** ^1^ Department of Pathogen Biology, School of Basic Medicine, Southwest Medical University, Luzhou, China; ^2^ Department of Oral prosthodontics, The Affiliated Stomatological Hospital of Southwest Medical University, Luzhou, China; ^3^ Department of Microbiology, Tumor and Cell Biology, Karolinska Institutet, Stockholm, Sweden; ^4^ Department of Dermatology, The First Affiliated Hospital of Southwest Medical University, Luzhou, China; ^5^ Department of Laboratory Medicine, Jiangnan University Medical Center, Wuxi, China; ^6^ Public Center of Experimental of Pathogen Biology Platform, Southwest Medical University, Luzhou, China

**Keywords:** hospital sewage, bla_VIM-2_, transposon, tigecycline resistance gene cluster, *Pseudomononas asiatica*

## Abstract

**Introduction:**

Tigecycline and carbapenems are considered the last line of defense against microbial infections. The co-occurrence of resistance genes conferring resistance to both tigecycline and carbapenems in *Pseudomononas asiatica* was not investigated.

**Methods:**

*P. asiatica* A28 was isolated from hospital sewage. Antibiotic susceptibility testing showed resistance to carbapenem and tigecycline. WGS was performed to analyze the antimicrobial resistance genes and genetic characteristics. Plasmid transfer by conjugation was investigated. Plasmid fitness costs were evaluated in *Pseudomonas aeruginosa* transconjugants including a *Galleria mellonella* infection model.

**Results:**

Meropenem and tigecycline resistant *P. asiatica* A28 carries a 199, 972 bp long plasmid PLA28.4 which harbors seven resistance genes. Sequence analysis showed that the 7113 bp transposon Tn*7389* is made up of a class I integron without a 5’CS terminal and a complete *tni* module flanked by a pair of 25bp insertion repeats. Additionally, the Tn*7493* transposon, 20.24 kp long, with a complete 38-bp Tn*1403* IR and an incomplete 30-bp Tn*1403* IR, is made up of partial skeleton of Tn*1403*, a class I integron harboring *bla*_OXA-10_, and a Tn*5563*a transposon. Moreover, one *tnfxB3-tmexC3.2-tmexD3b-toprJ1b* cluster was found in the plasmid and another one in the the chromosome. Furthermore, plasmid PLA28.4 could be conjugated to *P. aeruginosa* PAO1, with high fitness cost.

**Discussion:**

A multidrug-resistant plasmid carrying *tmexCD3-toprJ1b* and two novel transposons carrying *bla*_VIM-2_ and *bla*_OXA-10_ -resistant genes was found in hospital sewage, increasing the risk of transmission of antibiotic-resistant genes. These finding highlight the necessary of controlling the development and spread of medication resistance requires continuous monitoring and management of resistant microorganisms in hospital sewage.

## Introduction

Tigecycline is one of the last lines of defense against carbapenem-resistant bacterial infections (Aghapour et al., 2019; Jo and Ko, 2021). The Resistance-Nodulation-Division (RND) MDR efflux pump gene cluster *tmexCD1-toprJ1* or the variants such as the *tnfxB3-tmexC3.2-tmexD3b-toprJ1b* is one of the mechanisms which mediates the tigecycline resistance. Additionally, metallo-*β*-lactamases (MBLs) and carbapenemase coding genes like *bla*_KPC_ are the main mechanisms mediating carbapenem resistance (Hu et al., 2021; Huang et al., 2022). Emergence of tigecycline and carbapenem resistant bacteria such as *E. coli, Klebsiella* spp. and the *Pseudomonas* spp. from the patients poses great challenges to infection control (Lv et al., 2020; Wang et al., 2021a; Wang et al., 2021b; Gao et al., 2022; Li et al., 2022).

The genes encoding resistance determinants such as MBLs are usually found in plasmids or are associated with integrons and transposons (Mann et al., 2022). Integrons are able to capture genes that are part of gene cassettes *via* a site-specific recombination event and transposons contribute significantly to the transfer and transmission of antibiotic resistance (AR) in bacterial populations (Alavi et al., 2011; Mann et al., 2022). It is commonly believed that the hospital sewage provides a significant platform for the generation of new transposons and many of the novel transposons have been reported from the sewage. *Acinetobacter johnsonii* M19 isolated from hospital sewage carries a novel transposon Tn*6618* containing carbapenem resistant gene *bla*_OXA-23_, while *Shewanella xiamenensis* T17 carries the novel transposon Tn*6297* encoding OXA-416 (Yousfi et al., 2017; Zong et al., 2020).

*P. asiatica*, a newly proposed unique species of the genus *Pseudomonas*, belongs to the *Pseudomonas putida* group, which is a potential human pathogen that can cause nosocomial illness (Tohya et al., 2019a). Moreover, the most prevalent carbapenem resistance gene in the genome of clinical isolates of *P. aeruginosa* is the *bla*_VIM-2_ Metal -β -lactamase (MβL) gene, which is usually present in part of the cassette repertoire of class 1 integrons/transposons (Botelho et al., 2018). The *bla*_VIM-2_ gene has been found in *P. asiatica* (Brovedan et al., 2021; Tohya et al., 2021), indicating that it is an important reservoir of this gene.

Here, we describe a novel plasmid that co-harbors the tigecycline association resistance gene *tmexCD3-toprJ1b*, a *bla*_VIM-2_-carrying novel transposon Tn*7389*, as well as *bla*_OXA-10_-carrying novel transposon Tn*7493* from a *Pseudomonas asiatica* strain.

## Material and methods

### Bacterial isolation and identification

Wastewater samples were collected from a large tertiary hospital in Luzhou in August 2019. The sewage samples were collected from outflow of the sewage treatment stations of hospital. The samples were collected in sterile glass bottles (200ml) at a set time each time. Sewage samples were mixed and diluted with sterile water in a ratio of 1:10 and subsequently inoculated on a MacConkey agar plate at 37°C for 18-24h in the presence of antibiotics: meropenem (0.5 mg/L). One strain, named A28, was isolated and purified three times on Luria-Bertani (LB) broth agar medium following the repeated plate streaking method. The species was identified by detecting the 16S rRNA gene with universal primers 27F (5′-AGA GTT TGA TYM TGG CTC AG-3′) and 1492R (5′-GGY TAC CTT GTT ACG ACT T-3′), and further confirmed by WGS analysis (Smyth et al., 2020).

### Antimicrobial susceptibility test

The minimal inhibitory concentrations (MICs) of A28 to antimicrobial agents were determined by broth microdilution method according to the recommendations of the CLSI 2021 breakpoints. *Escherichia coli* strain ATCC 25922 was used as quality control.

### Whole-genome sequencing and analysis

The whole genome of strain A28 isolate was sequenced using Oxford Nanopore Technologies. The species was identified using JSpecies (http://jspecies.ribohost.com/jspeciesws/#analyse). ARGs were identified using ResFinder v.4.1 (https://cge.cbs.dtu.dk/services/ResFinder). MLST (Multi-Locus Sequence Typing) v.2.0 (https://cge.cbs.dtu.dk/services/MLST/) was used to determine the STs of the strain. RAST server v.2.0 (https://rast.nmpdr.org/rast.cgi) was used for genome annotation. The circular map of plasmids was generated using the BLAST Ring Image Generator (BRIG) tool and compared to highly similar plasmids in the NCBI database. The Transposon Registry assigned a name to the novel transposon (https://transposon.lstmed.ac.uk/).

### Conjugation assay and fitness cost of plasmid carriage

Conjugation assays were carried out using sodium azide-resistant *E. coli* J53, rifampicin-resistant *E. coli* EC600 (Rif^r^), and rifampicin-resistant *P. aeruginosa* PAO1 as recipients. Transconjugants were selected on LB agar plates containing meropenem (0.5 mg/L) and sodium azide (100 mg/L) or rifampicin (100 mg/L). The donor and recipient strains were mixed in ratios of 1:1, then cultured overnight on LB agar plates at 37°C.The resistance genes of *bla*_VIM-2_ in transconjugants were validated by PCR. A growth curve assay was used to calculate the fitness of the plasmid between *P. aeruginosa* transconjugants and *P. aeruginosa* PAO1 (Zhang et al., 2022). Overnight cultures were diluted 1:50 in LB without antibiotics and measured at OD_600_ every 15 minutes for 11 hours on a Synergy H1 (Labsystems) instrument, with each sample repeated three times. Student’s t-test was used for statistical analysis, with a significance threshold of 95% (*P*< 0.05).

### Biofilm formation

The ability of the transconjugant and wild-type strain to generate biofilms was determined using crystal violet staining (Ding et al., 2021). The bacterial suspension was discarded and washed three times with sterile water after standing culture at 37°C for 24 hours. Crystal violet was dissolved in a 33% acetic acid solution, and its OD_595_ value was determined.

### Galleria mellonella killing assay

By using serial dilutions, the transconjugant PAO1-A28 and *P. aeruginosa* PAO1 were divided into two different amounts of bacterial suspensions ranging from 1×10 ^5^ c.f.u. ml ^−1^ to 1×10 ^6^ c.f.u. ml ^−1^. Using a microsyringe, 10µl of the prepared bacterial suspensions were injected into the body cavity of *G. mellonella* through the right hind foot. The control group was injected with 10 µl PBS buffer. Ten *G. mellonella* were injected with bacteria in each group and placed in a Petri dish at 37° C for 72 hours. At 12-hour intervals, *G. mellonella* was observed to survive.

## Results

### Characterization of the strain *P. asiatica* A28

Bacterium A28 was identified as *P. asiatica* and was resistant to meropenem, imipenem, tigecycline, gentamicin, ceftazidime, aztreonam, and ciprofloxacin, but susceptible to polymyxin and tetracycline (**Table 1**). The genome of *P. asiatica* strain A28 was assembled into two complete circularized contigs, one chromosome (5824126 bp, CP063456.1) with GC content 62.51% and one plasmid PLA28.4 (199972 bp, CP063457.1) with GC content 56.36%. Species identification with ANI analysis confirmed that the strain A28 belonged to *P. asiatica*, A28 and had a 98.75% identity (89.30% query coverage) to *P. asiatica* RYU5 strain (accession: SAMN05581751) (Tohya et al., 2019b). MLST analysis revealed that the ST of strain A28 was ST15.

**Table 1:** Antibiotic susceptibilities of *P. asiatica* A28 and transconjugant (mg/L).

Strain	MIC (mg/L)										
	C	Gn	CAZ	TET	AZT	IPM	MEM	CIP	CTX	TIP	PB
*P. asiatica* A28	64	64	256	4	32	32	32	4	>128	4	0.25
*P. aeruginosa* PAO1	32	4	256	>512	8	8	1	<0.25	16	32	1
Conjugant PAO1-A28	32	32	256	>512	8	16	4	2	16	32	0.5

C, Chloramphenicol; Gn, Gentamicin; CAZ, Ceftazidime; TET, Tetracycline; AZT, Aztreonam; IPM, Imipenem; MEM, Meropenem;CIP, Ciprofloxacin; CTX, Cefotaxime; TIP, Tigecycline; PB, Polymyxin B.

### Characterization of plasmid PLA28.4

**Figure 1:**
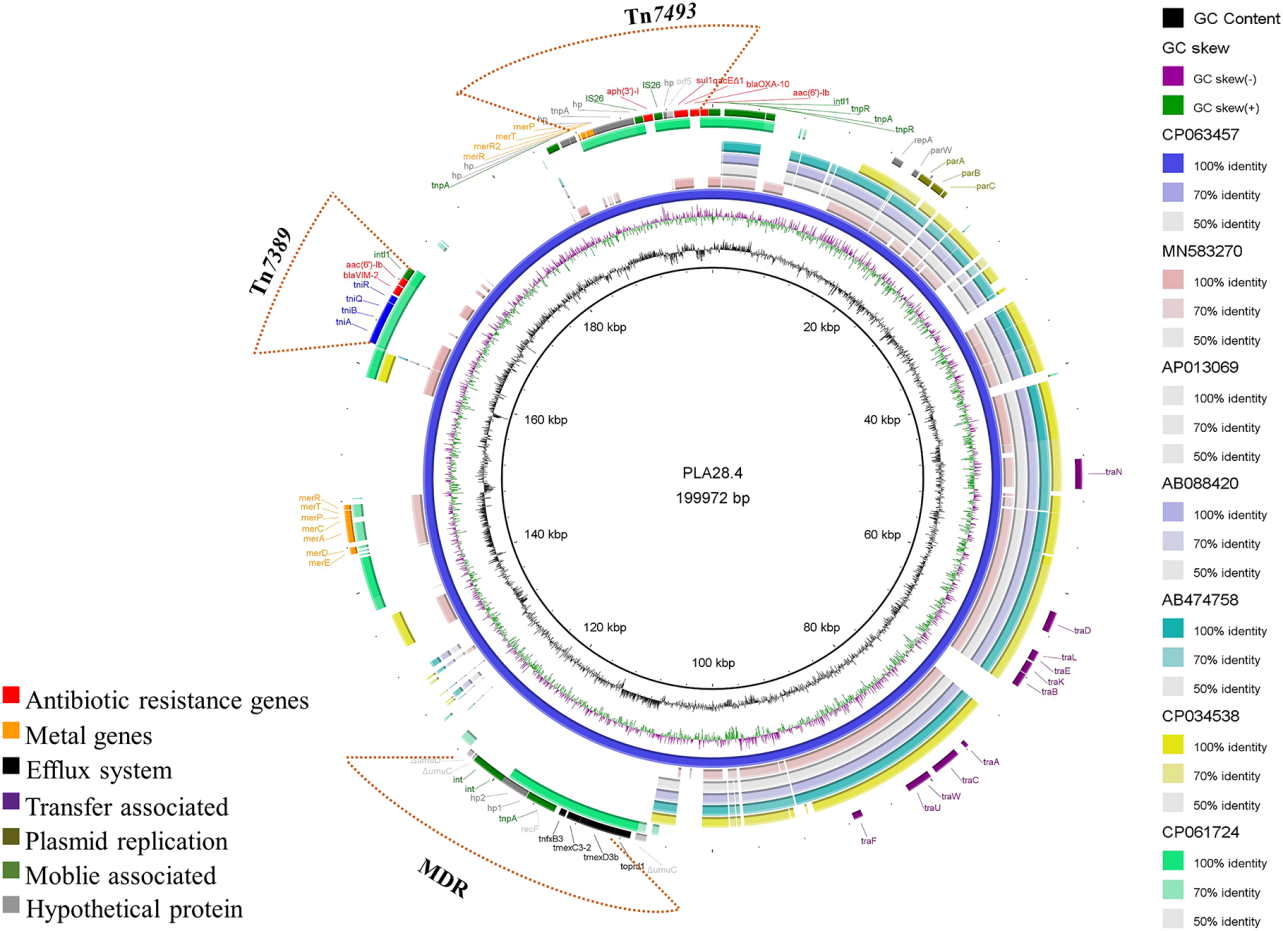
Comparative structural analysis of PLA28.4 with other similar plasmids available in the NCBI nr database. Starting from the center: (1) GC content of PLA28.4 with an average of 56.36%. (2) GC skew, with a positive GC skew toward the inside and a negative GC skew toward the outside. (3) The reference plasmid PLA28.4 plasmid sequence (CP063457). (4) Plasmid pNK546b (MN583270). (5) Plasmid pCAR1.3 (AP013069). (6) Plasmid pCAR1 (AB088420). (7) Plasmid pCAR1.2 (AB474758). (8) Plasmid unnamed (CP034538). (8) Plasmid pZXPA-20-602k (CP061724). (9) Gene annotation. The Figure was constructed using BRIG.

Plasmid PLA28.4 is a 199,972 bp circular plasmid with 233 predicted open reading frames. PLA28.4 does, however, feature a putative replication initiator protein RepA (encoded by bp16,426 to 17,292) that has 100% cover and 93.43% amino acid sequence similarity to RepA from the IncP-7 plasmid pCAR1 (GenBank accession number AB088420.1) in *P. resinovorans* (Maeda et al., 2003). ParA (encoded by bp 18444 to 18923) and ParB (encoded by bp 19123 to 20256) are partitioning proteins that are 80.62% to 97.07% similar to the partition proteins of the IncP-7 plasmid pCAR1. Besides, plasmid PLA28.4 carried 7 resistance genes, including *bla*_VIM-2_, *bla*_OXA-10_, *aac(6’)-Ib3*, *aph(3’)-I*, *sul1*, *aac(6’)-Ib-cr*) and the RND-type efflux pump gene cluster tnfxB3-tmexC3.2-tmexD3b-toprJ1b (**Figure 1**).

Sequence analysis showed that PLA28.4 was closely related to the IncP-7 plasmid Pnk546b (GenBank accession number MN583270.1) (Li et al., 2020) with a query coverage rate of 50% and identification rate of 84.74%. Additionally, PLA28.4 shares a similar plasmid backbone with the IncP-7-type plasmid pCAR1.3 (GenBank accession number AP013069.1) and pCAR1 (Shintani et al., 2006) from *Pseudomonas resinovorans*, and an unnamed plasmid (GenBank accession number CP034538.1) from *Pseudomonas poae*. Including the replication/partition region *repA-parW-parA* as well as the conjugative transfer system (consisting of *traNDLEKBACWUF* mobility genes), indicating that the plasmid PLA28.4 is conjugative.

Moreover, plasmid PLA28.4 had 23% sequence coverage and 99.27% identity with the megaplasmid pZXPA-20-602k (GenBank accession number: CP061724.1) from *P. putida*, which has both *bla*_VIM-2_ and multidrug resistance efflux pump *TmexCD1-ToprJ1*-like gene cluster (Li et al., 2021).

### Identification of the novel transposon Tn*7389*

**Figure 2:**
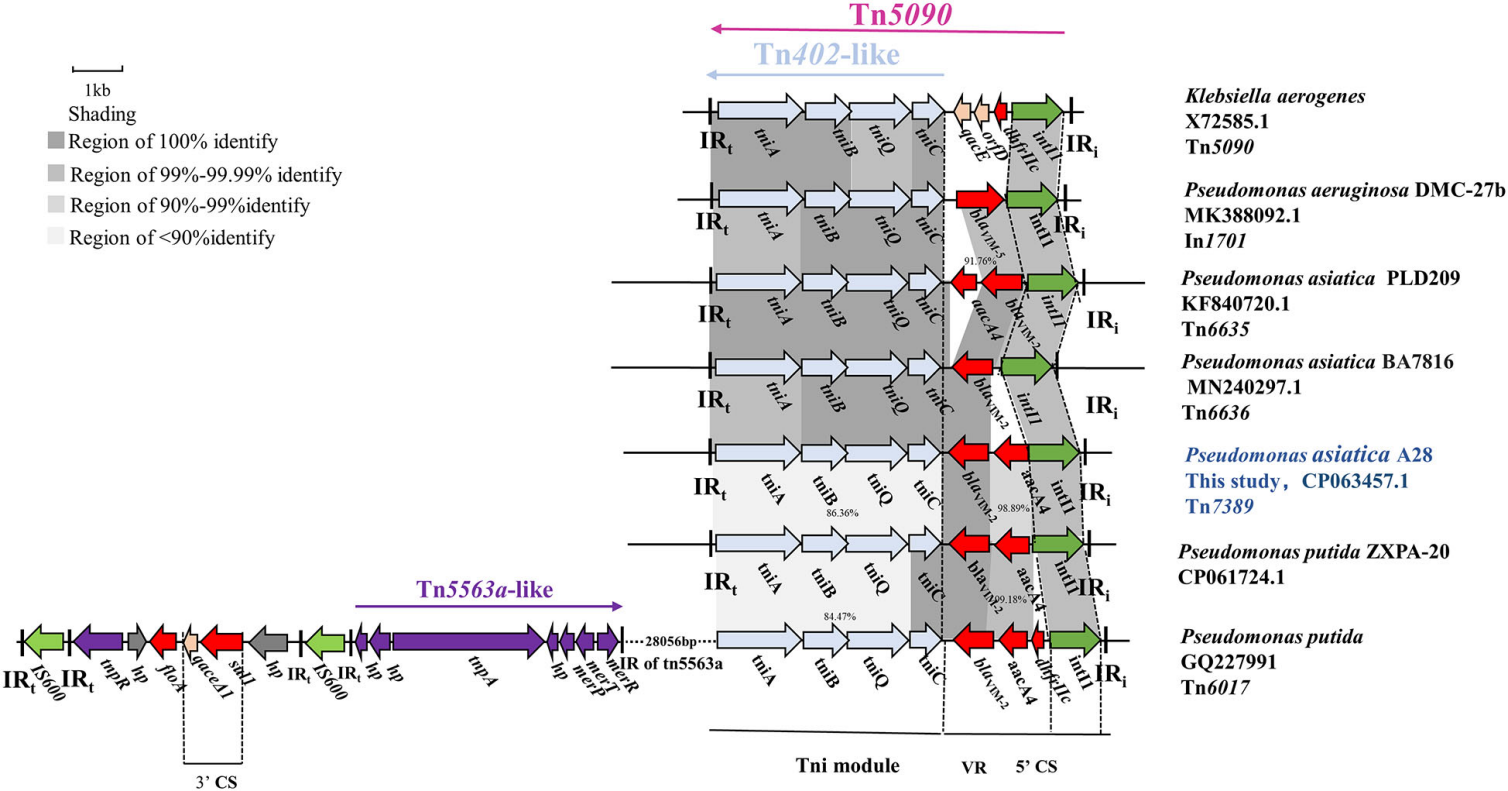
Genetic environment of the novel Tn*402*-like transposon Tn*7389* in *P. asiatica* A28. The construction of sequence comparison was performed using BLAST (http://blast.ncbi.nlm.nih.gov). Green arrows, integrases of a class of integrons; Light blue arrow: Tn*402* tni module; red arrows, antibiotic resistance genes; purple arrows, Tn5563a-like genes; gray arrows, hypothetical protein.

Tn*7389* is a new transposon with a 7113 bp backbone and three accessory modules. A complete Tn*402*-like tni module showed 99.98% nucleotide sequence similarity with the genes for transposase (*tniA*), transposase helper proteins (*tniB*, *tniQ*) and decomposition enzymes (*tniC*) of *Klebsiella aerogenes* Tn*5090* (Encoding a consistent sequence of corresponding proteins). The 5’ CS of Tn*7389* is an incomplete class 1 integron that lacks the 3’ CS and contains the antibiotic resistance gene cassette(*aacA*4-*bla*_VIM-2_) and lacks the 3’ CS (**Figure 2**). Tn*7389* differs from the In*1701* gene cassettes found on *P. aeruginosa* DMC-27B, and their integrase is one base inconsistent (Jahan et al., 2020). Tn*7389* has two resistance genes, *bla*_VIM-2_ and *aacA4*, but In*1701* only has one carbapenem resistance gene, *bla*_VIM-5_. Tn*402*-like transposons Tn*6635* and Tn*6636* harboring *bla*_VIM-2_ were also discovered in two *P. asiatica* strains, and these two transposons carried the same entire Tn*402*-like tni module, but only the *tniA* gene had one base mutation (G409A) compared to Tn*7389* (Brovedan et al., 2021). Tn*7389* has the same structure as the Tn*6017* transposon found in *P. aeruginosa* and *P. putida* isolated from a Spanish hospital (Juan et al., 2010). However, the similarity of tni modules is only 86.36%. Tn*7389* displayed an inconsistent arrangement of resistance genes on the gene cassettes compared to the Tn*402*-like transposon on the plasmid of *P. asiatica* LD209 (Marchiaro et al., 2014). Compared to the megaplasmid PZXPA-20-602K, Tn*7389*’s variable region (VR) lacks the *dhfrIIc* gene, whereas the Tn*5090*-like transposon of PZXPA-20-602K has a complete type 1 integron 3’ CS region with a size of more than 46 kbp (Li et al., 2021).

### Identification of the novel transposon Tn*7493*

**Figure 3:**
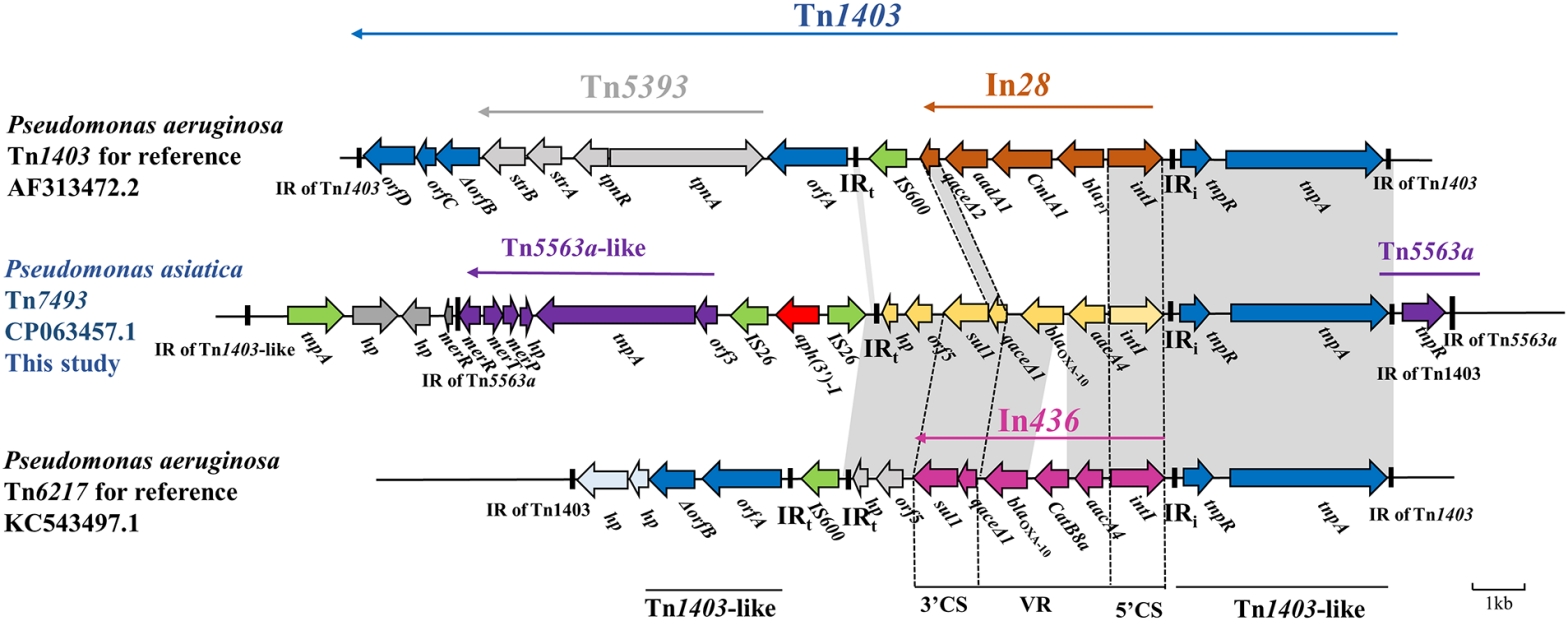
Genetic environment of the novel Tn*1403*-like transposon Tn*7493* in *P. asiatica A28*. The extents and directions of genes are shown by arrows labeled with gene names. The construction of sequence comparison was performed using BLAST (http://blast.ncbi.nlm.nih.gov).

The *bla*_OXA-10_ gene locates within a compound Tn*1403*-like transposon of 20.24 kp length, flanked by a complete 38-bp IR of Tn*1403* and an incomplete 30-bp IR of Tn*1403*, and was named Tn*7493* (**Figure 3**). Two cassettes, *aacA4*-*bla*_OXA-10_, encoding resistance to aminoglycosides and oxacillinase, were found in the class 1 integron. Upstream gene cassettes were 5’ CS of *intI1* and IRi, flanked by *tnpAR* and 38-bp IR of transposon Tn*1403* (Stokes et al., 2007), and *tnpR*, 39-bp-long IRs of Tn*5563a*. Downstream of *aacA4*-*bla*_OXA-10_ was *sul1*-type 3’ -CS, *orf5-hp*, and IRt, almost identical to the transposon Tn*6217* reported from *P. aeruginosa* (Xiong et al., 2013). On the flanks of IRt were two reverse insertion sequences, IS*26*, with an *aph(3’)-I* gene in the middle. The 3’ CS is a truncated transposon Tn*5563a* that contains a mercury resistance operon (*merPTR*) (Szuplewska et al., 2014), without the 3’ CS of Tn*1403* and Tn*5393.*

### Identified the tmexC3.2- tmexD3b-toprJ1b in P. asiatica A28

**Figure 4:**
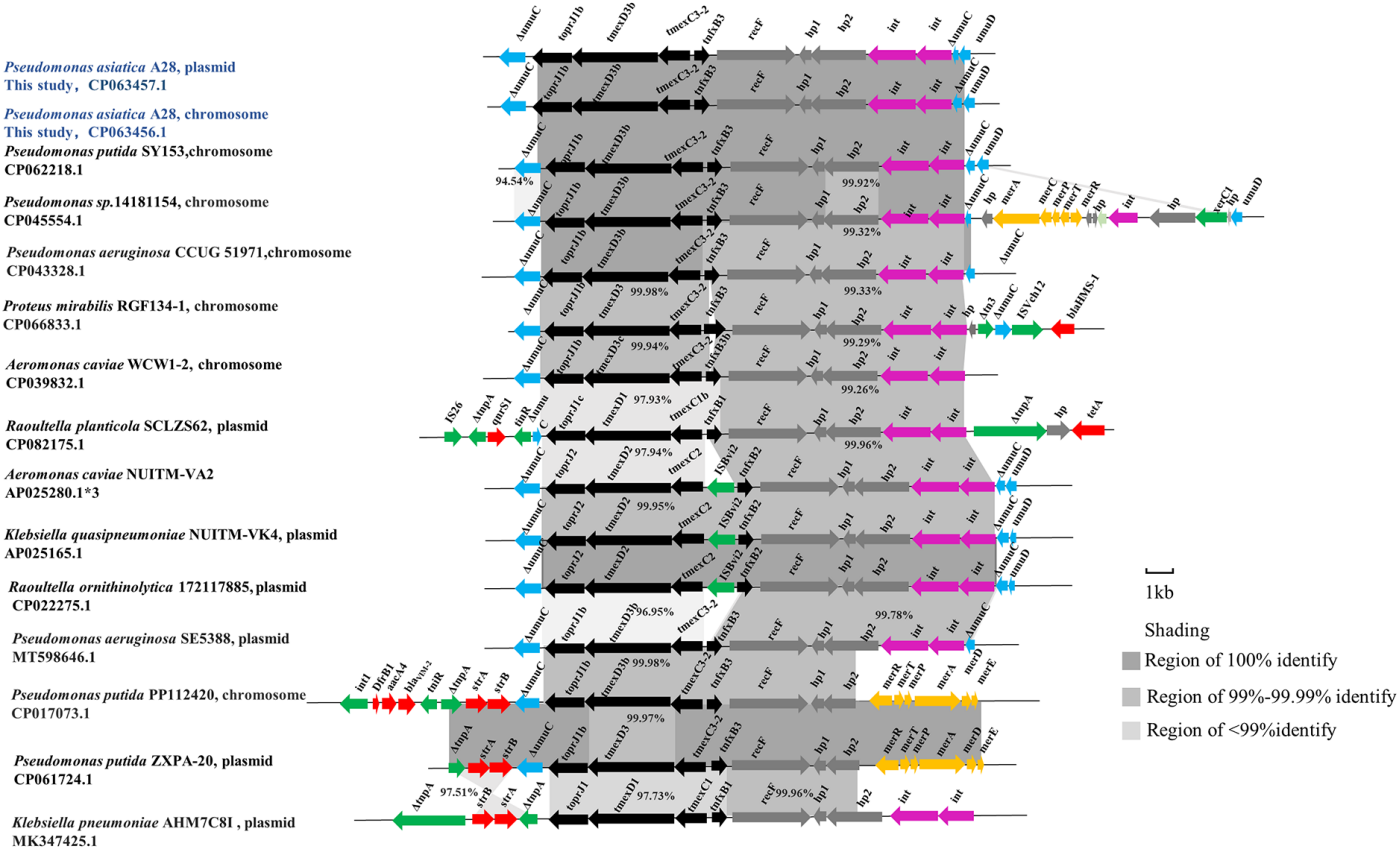
The genetic context of the multidrug resistant efflux pump *tnfxB3-tmexC3.2-tmexD3b-toprJ1b*. The extents and directions of genes are shown by arrows labeled with gene names. Black arrows, *tnfxB1-tmexCD1-toprJ1*-like gene clusters; pink arrows, int and int-like genes, predicted to encode site-specific integrases; blue arrows, *umuC* and *umuD*; green arrows, mobile related genes; red arrows, antibiotic resistance genes; yellow arrows, mercury resistance genes; gray arrows, hypothetical protein. Regions of homology between 96% and 100% are shaded.

Two identical RND-type efflux pump fragments *tnfxB2-tmexC3.2-tmexD3b-toprJ1b* coexist in the chromosome and plasmid PLA28.4 of *P. asiatica* A28 (**Figure 4**). The *tnfxB2-tmexC3.2- tmexD3-toprJ1b* fragment was 100% identical to the cluster found in other six *Pseudomonas* spp. from *Homo sapiens*. (GenBank accession no. CP045554.1, CP039989.1, CP017073.1, CP064948.1, CP064945.1, CP062218.1) and 99.98% identical (one nucleotide substitution) to another cluster found in *P. putida* megaplasmid pZXPA-20-602k (GenBank accession number: CP061724.1) (from a migratory bird, Zhejiang, China) (Li et al., 2021). Like the *tnfxB2-tmexCD1-toprJ1* cluster of *K. pneumoniae* AHM7C8I (Lv et al., 2020) (GenBank accession number: MK347425.1), *tnfxB3* ***-*** *tmexC3.2-tmexD3b-toprJ1b* is adjacent to *recF* (encoding AAA family ATPase), two hypothetical genes *(hp1* and *hp2*), and two site-specific integrase genes (*int1* and *int2*). Of these, *recF* has a single base substitution (A2283G), and *hp2* has one base substitution (G1820T). *P. asiatica* A28 had 100% similarity with the *tnfxB3-tmexC3.2-tmexD3b-toprJ1b-recF-hp1-hp2-int1-int2* structure of *P. aeruginosa* (GenBank accession no. CP039989.1) and *P. putida* (GenBank accession no. CP062218.1).

### Conjugation assay, fitness cost, biofilm formation, and *G. mellonella* killing assay

**Figure 5:**
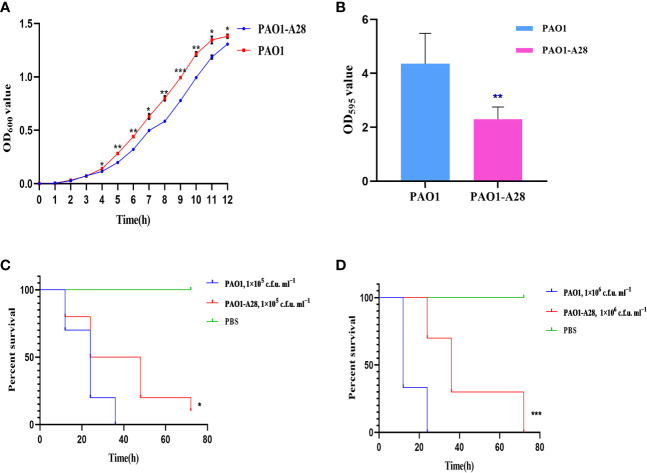
Fitness costs and stability of PLA28.4 in strain *P. aeruginosa* PAO1. **(A)** Growth curve of the transconjugant and recipient PAO1. **(B)** Biofilm formation of the transconjugant and recipient PAO1. **(C, D)** Survival of *G. mellonella* following infection with the transconjugant and recipient PAO1. *Statistically significant (p < 0.05), **statistically significant (p < 0.01), and ***statistically significant (p < 0.001).

The plasmid PLA28.4 could not be transferred to the recipient cell *E. coli* J53/C600 by conjugation but could be transferred to *P. aeruginosa* PAO1. The transfer frequency of PLA28.4 was (2.039±0.077) × 10^-8^ per recipient. Consequently, we evaluated the effect of acquiring resistance plasmids on biological fitness and observed significant differences in growth rate related to plasmid acquisition in *P. aeruginosa* PAO1 from 4h-12h (P <0.0001, **Figure 5A**). Biofilm formation was significantly reduced in the transconjugant strain (P<0.05) (**Figure 5B**). We examined the susceptibility of *G. mellonella* to the transconjugant PAO1-A28 and *P. aeruginosa* PAO1, which were injected with 1×10 ^5^ c.f.u. ml ^−1^ to 1×10 ^6^ c.f.u. ml ^−1^ of the strains and incubated in the dark at 37°C for up to 72 h. As shown in **Figures 5C, D**, compared with PAO1, the transconjugant PAO1-A28 showed significantly reduced virulence against *G. mellonella* (P<0.05). The decreased virulence of transconjugant to *G. mellonella* might be due to the adaptive cost of plasmids.

## Discussion

As an important reservoir of ARB and ARG, hospital sewage is an important medium for ARG to spread to other environments. In this study, a tigecycline and carbapenem-resistant culture obtained from hospital sewage belonged to *P. asiatica* ST15, which is a newly proposed unique species of the genus *Pseudomonas*, belongs to the *Pseudomonas putida* group (Tohya et al., 2020). Sequencing analysis revealed that it coharboring carrying a *tmexCD3-toprJ1b*, a novel Tn*5090*-like transposon Tn*7389* harboring *bla*_VIM-2_, and a Tn*1403*-like transposon Tn*7493* harboring *bla*_OXA-10_. Tn*5090* (also known as Tn*402*) was discovered on IncP-7 plasmid R751 from *K. aerogenes* in 1994 (Rådström et al., 1994). In Tn*7389*, two 25-bp initial reverse repeat (IRi) and terminal reverse repeat (IRt) of Tn*5090*/Tn*402* transposon families were located 171 bp downstream of *intI1* and 116bp upstream of *tniA*, respectively, suggesting that the *bla*_VIM-2_ could be mobilized using the tni machinery. The integrase and recombination sites containing class 1 integrons can be inserted and removed in the form of gene cassettes at *attI*1 (Toleman and Walsh, 2011). Multiple Tn*5090*-like transposons carrying *bla*_VIM-2_ have been found in *Pseudomonas* in a growing number of investigations, suggesting that Tn*5090*-like transposons are key mobile components of VIM-2 transmission in *Pseudomonas* (Santos et al., 2010). The *bla*_VIM-2_ gene could be mobile *via* the tni mechanism, which may promote its transmission among other pathogens in the hospital sewage environment and requires closer monitoring.

Tn*1403* was discovered on RPL11 plasmids from clinical *P. aeruginosa* isolates expressing resistance to ampicillin, streptomycin, puromycin, and chloramphenicol (Vézina and Levesque, 1991). Tn*1403*-like transposons have been found primarily in *Pseudomonas* spp. and have been shown to carry diverse types of ARGs, suggesting that they may play an important role in ARG and metal resistance gene transmission in *Pseudomonas.* In addition, disinfectant-sulfanilamide resistance (*qacEΔ*1-*sul*1) genes cause bacterial resistance to chlorine-containing disinfectants and allows bacteria to survive in disinfected water, which poses a threat to health care systems.

Although there are different variants of the MDR efflux pumps *tmexCD1-toprJ1*, similar structures have also been found in *Aeromonas caviae*, *Raoultella planticola*, and *Klebsiella quasipneumoniae*, suggesting potential horizontal transfer mechanisms among various species (Wang et al., 2021a; Dong et al., 2022; Gao et al., 2022). The transfer of *tnfxB2-tmexCD1-toprJ1* has previously been found to be mobilized by site-specific integrase (Lv et al., 2020). However, it could be linked to *umuCD*, a neighboring mutant DNA repair system, because integrase can accelerate the excision and integration of *umuCD* (Peng et al., 2021). The proximity of *umuCD* to the efflux pump structure in various bacteria revealed that it might help spread *tmexCD1-TopRJ1*-like gene clusters.

The IncP-7 plasmid is a conjugative transfer plasmid with a narrow host range (Shintani et al., 2010). Although most reports suggest that IncP-7 plasmids could only be transmitted in *Pseudomonas* (Xiong et al., 2013), pCAR1 was discovered to be transferable to *Sterotrophomonas*-like strains in natural water (Shintani et al., 2008). Moreover, the IncP-7 type plasmid pNK546b in *P. aeruginosa* NK546 also assisted the transmission of another resistant plasmid pNK546a that could not be self-transmissible (Li et al., 2020). In this study, the IncP-7 plasmid PLA28.4 of *P. asiatica* could be transferred to *P. aeruginosa* PAO1, suggesting PLA28.4 has the capacity to transmit numerous resistance genes in hospital sewage, according to this study. Collectively, plasmid fitness cost studies found that transferring the PLA28.4 plasmid into *P. aeruginosa* PAO1 resulted in a lower growth rate, less biofilm generation, and lower pathogenicity, demonstrating that transmission of the PLA28.4 plasmid caused bacteria to pay a cost of adaptation.

We discovered a *P. asiatica* carrying a plasmid containing the *tmexCD1-toprJ1*-like gene cluster, and two novel transposons carrying *bla*_VIM-2_ and *bla*_OXA-10_, respectively. Controlling the development and spread of medication resistance requires continuous monitoring and management of resistant microorganisms in hospital sewage.

## Conclusion

We discovered a *P. asiatica* carrying a plasmid containing the *tmexCD1-toprJ1*-like gene cluster, and two novel transposon carrying *bla*_VIM-2_ and *bla*_OXA-10_, respectively. Controlling the development and spread of medication resistance requires continuous monitoring and management of resistant microorganisms in hospital sewage.

## Data availability statement

The datasets presented in this study can be found in online repositories. The names of the repository/repositories and accession number(s) can be found below: https://www.ncbi.nlm.nih.gov/genbank/, CP063456.1.

## Author contributions

SL, WW, BYZ and QHL collected the data. FYZ and LG performed the bioinformatic analyses. QL, QC, AM-R wrote the initial draft of the manuscript. QL, RJH, LX and YSZ conceived the project, reviewed the articles and extracted the data. XX contributed to the revision of this article. All authors contributed to the article and approved the submitted version.
